# Development of serologic diagnostic test based on *in silico* predicted synthetic peptides for *Brucella canis* in dogs

**DOI:** 10.1371/journal.pone.0342574

**Published:** 2026-02-17

**Authors:** Monique Ferreira Silva Souza, Lucas dos Reis de Souza, Pamela Aparecida Lima, Tatyane Martins Cirilo, Samuel Alexandre Pimenta Carvalho, Lilian Lacerda Bueno, João Luís Reis Cunha, Tatiane Alves da Paixão, Ricardo Toshio Fujiwara, Renato Lima Santos

**Affiliations:** 1 Departamento de Clínica e Cirurgia Veterinárias, Escola de Veterinária, Universidade Federal de Minas Gerais, Belo Horizonte, Minas Gerais, Brazil; 2 Departamento de Patologia Geral, Instituto de Ciências Biológicas, Universidade Federal de Minas Gerais, Belo Horizonte, Minas Gerais, Brazil; 3 Departamento de Parasitologia, Instituto de Ciências Biológicas, Universidade Federal de Minas Gerais, Belo Horizonte, Minas Gerais, Brazil; 4 University of York, York, United Kingdom; UFPL, BRAZIL

## Abstract

Serologic diagnosis of canine brucellosis caused by *Brucella canis* remains quite challenging since the currently available methods have considerable limitations, although the relevance and awareness about this zoonotic pathogen is increasing over the past few years. Therefore, the development of novel strategies is highly desirable. In this study, *in silico* analyses resulted in prediction of *B. canis* specific B cell epitopes, which were validated by synthesizing the corresponding peptides on a membrane followed by immunobloting with sera from dogs naturally infected with *B. canis* or uninfected controls as well as by excluding epitopes that cross reacted with sera from cattle and sheep infected with *B. abortus* and *B. ovis*, respectively. This approach resulted in the identification of 26 epitopes that reacted exclusively with sera from dogs infected with *B. canis*, from which the 15 with strongest signal in the immunoblot were selected and synthesized in soluble form for further analyses. The best 10 synthetic peptides (considering the noise to signal ratio) were used in combination as antigens for development of a *B. canis* specific indirect enzyme-linked immunosorbent assay (iELISA) protocol, which yielded improved specificity to differentiate from other common canine pathogens (*Leishmania* sp. and *Babesia* sp.) and an improved performance (100% specificity and 80% sensitivity) when compared to crude bacterial protein extracts used as antigen for iELISA. These selected epitopes were also incorporated in a multi-*B. canis* epitope protein that was expressed in *E. coli* and employed as antigen for detection of anti-*B. canis* IgG and IgM from naturally infected dogs, resulting in analytical performance similar to the iELISA protocol in which synthetic peptides were used as antigens (100% specificity and 75% sensitivity). The results clearly indicate that combination of detection of IgM and IgG may result in higher sensitivity. Therefore, this study provides novel tools for improving the accuracy of serologic diagnosis of canine brucellosis caused by *B. canis*.

## Introduction

Brucellosis is a major zoonotic disease with worldwide distribution, caused by many *Brucella* species with variable clinical manifestations depending on the host species. Importantly, in spite of taxonomic controversies, most of the *Brucella* species have zoonotic potential causing human disease [1]. In dogs, brucellosis is more commonly caused by *Brucella canis*, a species that, despite being less pathogenic to humans, represents an important zoonotic risk due to the proximity between humans and dogs [2]. The prevalence of canine brucellosis is considered underestimated due to difficulties in diagnosis [3] and the high frequency of asymptomatic dogs [4,5]. In dogs, infection by *B. canis* is related to reproductive alterations, such as fetal and neonatal mortality [5,6], although *B. canis* is capable of infecting many tissues and organs of neonatal dogs in a pantropic fashion [5,7].

Many laboratory techniques have been developed for diagnosis of brucellosis in various host species [8]. However, the diagnosis of canine brucellosis is quite challenging, and usually requires combination of more than one diagnostic method, including indirect serologic methods as well as direct methods such as bacterial isolation and polymerase chain reaction (PCR) [2,3]. Serological tests include agar gel immunodiffusion (AGID) usually using *Brucella ovis* antigens for serological diagnosis of *B. canis* since both species have rough LPS, complement fixation, microagglutination test, and various indirect enzyme-linked immunosorbent assay (iELISA) protocols. Although useful, these tests have important limitations, such as low specificity, cross-reactions, and variation in performance according to the antigen used, leading to nonspecific reactions [3,4]. For instance, AGID has a diagnostic sensitivity of 52.94% and specificity of 100% [9]. Given these limitations, novel and more accurate diagnostic tools are highly desirable. Considering the role of B cells in the generation of a humoral immune response, the use of bioinformatics for B cell epitope prediction, followed by experimental validation, makes it possible to identify promising candidates for more accurate diagnoses. In this context, soluble synthetic peptides have emerged as promising antigen candidates in serological tests, offering advantages such as specificity and reproducibility as well as improved stability and biosafety (since no biohazard is involved in its production) when compared to full protein antigens. Target selection by bioinformatics, followed by spot synthesis technique, enables the simultaneous evaluation of different sequences of immunogenic synthetic peptides of bacterial proteins in cellulose membrane. This approach has already been successfully employed in other infectious diseases, such as leishmaniasis and tuberculosis (Guedes et al., 2019; López-Pérez et al., 2017; Zhang et al., 2014). *In silico* prediction of epitopes have also been recently applied for the development of diagnostic methods for brucellosis ) [10–13] as well as for *Brucella* vaccinology [14,15].

Thus, the present study aimed to develop and evaluate soluble synthetic peptides for specific detection of anti-*B. canis* using an indirect ELISA protocol for serological diagnosis of canine brucellosis.

## Materials and methods

### Ethics statement

Serum samples employed in this study were from our archive and were obtained in previous studies [3,5,16] that had protocols approved by the Institutional Animal Care and Use Committee (Comissão de Ética no Uso de Animais/Universidade Federal de Minas Gerais – CEUA/UFMG – protocols # 204/2010; 197/2014; 56/2016; and 365/2018).

### Serum samples

Previously characterized serum samples from natural hosts (cattle, dogs and sheep) were used for evaluating analytical suitability of *in silico*-predicted epitopes (Table 1). *Brucella* sp.-positive sera were from experimentally infected or naturally infected with *Brucella* sp., confirmed by direct methods (bacterial isolation or PCR). Negative controls included sera from non-infected animals with negative serologic and bacterial isolation or PCR.

**Table 1:** Serum samples employed in this study.

Animal	n**	Group	Brucellosis test applied to each sample*						Reference/ origin****
**RBT/** **2-ME**	**iELISA** ***	**AGID**	**MAT**	**PCR**	**Bacterial isolation**
Dog	10	Negative control for canine brucellosis	**–**	**–**	**–**	**–**	**–**	**–**	[3]
Dog	10	Positive for brucellosis (natural infection – *B. canis*)	**–**	**+**	**+**	**+** (n = 4) **–** (n = 6)	**+**	**+**	[3]
Dog	9	Specificity control (natural infection – *Leishmania*)	NA	**–**	NA	NA	NA	NA	ICB/UFMG
Dog	4	Specificity control (natural infection – *Babesia*)	NA	**–**	NA	NA	NA	NA	ICB/UFMG
Sheep	10	Negative control for ovine brucellosis	**–**	**–**	**–**	NA	**–**	**–**	[16]
Sheep	10	Positive control (experimental infection – *B. ovis*)	**–**	**+**	**+**	NA	**+**	**+**	[16]
Cattle	10	Negative control for bovine brucellosis	**–**	NA	**–**	NA	NA	NA	UFMG
Cattle	10	Positive for brucellosis (natural infection – *B. abortus*)	**+**	NA	NA	NA	NA	NA	LFDA/MG

* Abbreviations: RBT/2-ME: rose Bengal test/2-mercaptoethanol; iELISA: indirect enzyme-linked immunosorbent assay; AGID: agar gel immunodiffusion; MAT: microagglutination test; PCR: polymerase chain reaction; NA: not applicable.

** The number of canine samples had a slight decrease since some samples ran out over the course of the experiments.

*** iELISA using whole crude extract of *B. canis* as antigen.

**** ICB/UFMG: Instituto de Ciências Biológicas/Universidade Federal de Minas Gerais; UFMG: Universidade Federal de Minas Gerais; LFDA/MG: Laboratório Federal de Defesa Agropecuária de Minas Gerais.

Possible cross-reactions were assessed by using sera from animals negative for *Brucella* spp. and serologically positive for common and relevant canine pathogens including *Leishmania* sp. (n = 9) and *Babesia* sp. (n = 4).

### *In silico* prediction of B cell epitopes

*In silico* prediction of B cell epitopes (synthetic peptides) was based on the genomic sequences and predicted proteomes of five *Brucella* species, namely *Brucella melitensis*, *B. ovis*, *B. canis*, *Brucella abortus*, and *Brucella suis* available at the NCBI (National Center for Biotechnology Information) website (https://www.ncbi.nlm.nih.gov). Predicted proteomes of the selected species were filtered to include only high-quality sequences, based on the following criteria: proteins with initial methionine codon (Met), presence of terminal stop codon, absence of indeterminate amino acids (“X”), absence of internal stop codon, and 100 or more amino acids as detailed in S1 Table. Predicted proteins were then screened for detection of B-cell epitopes using BepiPred with a cut-off of 1.3 [17] and IUpred with a cut-off of 0.5 [18] predictors. Predicted epitopes corresponded to peptides containing from 9 to 15 amino acids and with the highest prediction scores.

After the prediction of epitopes, two groups of peptides were selected: (1) predicted epitopes conserved across all *Brucella* species; (2) species-specific *B. canis* predicted epitopes. Selection of conserved epitopes was based on 100% nucleotide identity with at least 85% coverage across all *Brucella* species evaluated using BLAST (https://blast.ncbi.nlm.nih.gov/Blast.cgi). Species-specific epitopes, peptides where those exclusively found in each of the *Brucella* spp. The pre-selected epitopes in both groups were then analyzed. Peptides with poor conformational stability, sequences smaller than nine amino acids and/or repetitive cycles with more than seven identical amino acids were discarded.

### Peptide screening

In order to assess reactivity of predicted epitopes (synthetic peptides), they were synthesized and spotted on cellulose membranes using the ResPepSL/Automatic Spot Synthesizer (Intavis, Cologne, Germany). Pools of positive or negative serum samples from natural hosts (cattle, sheep, and dogs, positive or negative for *B. abortus*, *B. ovis*, or *B. canis*, respectively; Table 1) were then incubated with membranes containing the predicted epitopes (synthetic peptides). First, the membrane was blocked with 5% BSA and 4% sucrose in 1x PBS for 12–16 h at room temperature, under agitation (50 rpm). The membrane was then washed three times with the washing solution (PBS with 0.1% Tween 20) under agitation (50 rpm) for 10 min each wash. The membrane was incubated with the pool of either positive or negative samples for 2 h, with two dilutions (1:500 and 1:1000). After incubation, the membrane was washed again three times with the wash solution (PBS with 0.1% Tween 20), under agitation (50 rpm) for 10 min each. The membrane was then incubated for 1 h with a secondary anti-IgG antibody conjugated with peroxidase (Sigma-Aldrich, St. Louis, USA). The membrane was washed again three times with the wash solution (PBS with 0.1% Tween 20), under agitation (50 rpm) for 10 min each, and then incubated with luminol substrate (Luminata ForteTM Western HRP substrate – Merck, USA). The spots were detected using the Image Quant LAS 4000 (GE Healthcare Life Sciences) with an exposure time of 1 min.

After each assay, the membrane was submitted to a regeneration treatment for reuse. The membrane was immersed in N,N-dimethylformamide (DMF) three times for 10 min and incubated with a denaturing solution (8 M urea; 1% SDS) for 12–16 h. The following day, the membrane was twice immersed in denaturing solution for 30 min and washed with Milli-Q water for 2 min. Acid solution (55% ethanol; 10% glacial acetic acid, and 35% Milli-Q water) was added for 10 min, repeated two more times. The membrane was then washed again with Milli-Q water for 2 min and then washed twice in ethanol for 5 min each, dried, and stored at 4°C.

Identification of reactive synthetic peptides was performed by densitometry (Image J Software – Protein Array Analyzer). Spots were analyzed for their reactivity and compared to among various sera tested. Selection of target synthetic peptides was based on specificity and reactivity. Synthetic peptides showing reactivity with negative sera (values above the cut-off, defined as the mean of the negatives plus 2x the standard deviation) and with weakly reactive positive spots (values below the same cut-off) were excluded. The synthetic peptides selected were those with the strongest reactivity, showing values above the cut-off defined as the mean of the negatives plus 2 x or 3 x the standard deviations.

### Synthesis and characterization of soluble selected epitopes (synthetic peptides)

The soluble peptides were synthesized at a scale of 25 μmol using a ResPep SL automated synthesizer (Intavis AG, Cologne, Germany). The amino acids were activated with a 1:1 solution of Oxyma Pure (Merk, Darmstadt, Germany) and dissopropylcarbodiimide (Sigma-Aldrich, Missouri, USA). Activated amino acids were incorporated into TentaGel (Intavis AG, Cologne, Germany) or H-Rink Amide ChemMatrix (Sigma-Aldrich, Missouri, USA) resins. Fmoc deprotection was performed repeatedly until the synthesis of each peptide was completed using 25% 4-methylpiperidine (25% v/v in DMF). Synthetic peptides were then extracted from the resin through a treatment with a solution containing 92.5% trifluoroacetic acid, 2.5% water, 2.5% triisopropyl silane, and 2.5% beta-mercaptoethanol for 3 h under agitation. Synthetic peptides were precipitated with a cold solution of methyl tert-butyl, and then lyophilized. The mass/charge ratio (H^+^) of each synthetic peptide was confirmed by mass spectrometry using the MALDI/TOF Autoflex Speed equipment (Bruker Daltonics, Massachusetts, USA). Aliquots of 0.5 μL of concentrated synthetic peptide was mixed with 0.25 mL of a saturated matrix solution [10 mg/mL α-cyano-4-hydroxycinnamic (Aldrich, Milwaukee, WI) and 50% acetonitrile/0.1% trifluoroacetic acid]. Samples were then applied to an MTP AnchorChip TM 600/384 board (Bruker Daltonics, Massachusetts, USA) and left to dry at room temperature. Raw data were obtained in MALD/TOF Autoflex Speed using a positive/reflector mode controlled through the FlexControl 3.3 software. Calibration of the instrument was performed using reference peptides (Peptide Standard, Bruker Daltonics, Massachusetts, USA) and each spectrum was produced by accumulating data from 200 consecutive lasers of 127 shots. After confirming the synthesis of the peptides, standardization of serological tests using the iELISA technique was performed.

### Production of a multi-epitope protein

*Escherichia coli* Shuffle and BL21 (DE3) strains were used for multi-epitope protein expression after transformation with a construct based on the plasmid pET-28a(+)-TEV (Merck, USA). The construct was cloned using the *Nhe*I and *Xho*I restriction sites, and contained coding sequences for all 10 selected epitopes with GKGK (Glycine-Lysine-Glycine-Lysine) linkers (GenScript, Nanjing, China). The order of peptides was defined by the predicted conformation of the final protein based on the maximum linearity (assessed by the degree of structural disorder using the IUPred software) of all possible sequence arrangements and the maximum scores of antigenicity (B cell epitopes). Transformed bacteria were cultured on 2xYT agar supplemented with kanamycin (50 μg/mL) at 30ºC for Shuffle or at 37°C for BL21, for 16–18 h. Isolated colonies of each bacteria were inoculated into 3 mL of 2xYT broth with kanamycin and incubated at 37°C (BL21) or 30ºC (Shuffle) at 180 rpm for 16 h (pre-inoculum). The final inoculum was prepared by diluting the pre-inoculum in a 1:20 ratio, followed by incubation under the same conditions until OD_600_ reached 0.6 to 0.8. One sample was obtained before induction (time 0 h) with IPTG (1 mM). Three h after induction, another sample was obtained (time 3 h), cultures were then centrifuged at 2,000 × g, and bacterial pellets were stored at –80°C until analysis.

Cell pellets containing the multi-epitope protein were ressuspended in 1 mL of PBS 1X buffer (Invitrogen, Thermo Fisher Scientific, USA), 10 μL of lysozyme (10 mg/mL; Sigma-Aldrich, USA) were added, followed by vortexing and incubation on ice for 30 min. The samples were then subjected to five cycles of freezing in liquid nitrogen and thawing at 37°C, interspersed with homogenization using a 1 mL insulin syringe (BD, Brazil), until the viscosity of the material was reduced. The lysate was centrifuged at 6,000 × g for 5 min at 4°C to separate the soluble (supernatant) and insoluble (pellet) fractions. In a fresh tube, 75 μL of the supernatant was mixed with 25 μL of 4X sample buffer (Laemmli Sample Buffer, Bio-Rad, USA) and identified as soluble fraction. The pellet was resuspended in 500 μL of PBS 1X and 75 μL of this suspension were mixed with 25 μL of 4X sample buffer and identified as insoluble fraction.

Soluble and insoluble fractions obtained at 0 h and 3 h post induction were denatured at 95°C for 10 min before loading on standard polyacrylamide gel followed by electrophoresis at 100–120 V. Gels were stained with Coomassie Brilliant Blue R-250 (Bio-Rad). Proteins in polyacrylamide gels were transferred to nitrocellulose membranes using 1X transfer buffer at 100 V for 1 h at 4^o^C. Membranes were blocked with Tris-buffered saline with 0.1% Tween 20 (TBS-T) 1X containing 5% skim milk. An anti-his primary antibody diluted 1:2000 in blocking solution were incubated overnight at 4ºC. Membranes were washed 3 times with TBS-T 1X and incubated with anti-IgG conjugated secondary antibody (1:5,000 dilution) for 1 h, under agitation. After final washing, proteins were detected using a chemiluminescent solution (Luminata; Merck, Darmstadt, Germany) and the development was performed in the ImageQuant LAS 4000 digital imaging system (GE Healthcare, Chicago, USA).

For larger scale expression, transformed *E. coli* strains were seeded on 2xYT agar supplemented with 50 μg/mL of kanamycin (Thermo Fisher Scientific, USA) and incubated at 37°C for 16–18 h for *E. coli* BL21 or at 30°C for 24 h for *E. coli* Shuffle. A single colony was inoculated in 50 mL of 2xYT broth containing 50 μg/mL of kanamycin and incubated at 37°C under agitation (180 rpm) for 16 h for BL21 or at 30°C for 24 h for *E. coli* Shuffle (pre-inoculum). Bacterial suspensions were then diluted 1:20 in 1 L of 2xYT broth supplemented with 50 μg/mL of kanamycin and incubated under agitation (180 rpm) at 37°C for BL21 or 30°C for *E. coli* Shuffle until optical density (OD₆₀₀) reached 0.6 to 0.8 (~3 h). Expression was induced with 1 mM of IPTG (Thermo Fisher Scientific, USA) for 3 h under the same conditions. Bacteria were collected by centrifugation at 2,000 × g for 30 min at 4°C (Eppendorf 5810R refrigerated centrifuge). Pellets were transferred to 50 mL Falcon tubes and stored at –80°C. Pellets were thawed on ice and resuspended in AKTA A buffer (PBS 1x + 30 mM Imidazole), in a ratio of 1:10 (v/v), i.e., 100 mL of buffer per liter of culture. Lysozyme (10 mg/mL; Sigma-Aldrich, USA) was added, followed by homogenization and incubation on ice for 1 h. Cell lysis was performed using a high-pressure EmulsiFlex homogenizer. Bacterial extract was transferred to autoclaved 500 mL flasks and centrifuged at 10,000 × g for 1 h at 4°C. The supernatant was filtered in a 0.45-μm membrane (Millipore, USA) and kept on ice. Protein was purified by affinity chromatography (AKTA Prime, GE Healthcare) by injecting 2 mL/min into the system. Absorbance (λ = 280 nm) was monitored. After stabilization, elution was initiated with B-buffer (PBS 1X + 500 mM midazole) at 1 mL/min per 20 mL, collecting fractions of 1.8 mL. Protein concentration was determined using the BCA Protein Assay Kit (Thermo Fisher Scientific, USA), according to the manufacturer’s instructions.

### iELISA

To determine the dilution of the serum samples, the concentration of synthetic peptides, crude antigen, and the dilution of secondary antibodies, and optimization of the ELISA protocol were performed (S2 Table).

The iELISA using whole-cell extracts of *B. canis* was performed coating plates with 250 ng of antigen per well, diluted (1:50) serum samples, and a secondary antibody goat anti-dog IgM (1:2,500 dilution) conjugated to peroxidase (Thermo Fisher, USA, Cat. No. [PA1–84638]) or a secondary antibody goat anti-dog IgG (1:2,500 dilution) conjugated to peroxidase (Invitrogen, USA, Cat. No. [PA1–29738]). ELISA plates (Corning Costar, USA) were sensitized with 100 μL of whole cells *B. canis* antigen for 18 h at 4ºC. Plates were washed three times with phosphate-buffered saline – PBS 1x, pH 7.2 with 0.05% Tween-20 (PBS-T) and blocked with 100 μL of PBS with 5% bovine serum albumin (BSA) for 1 h at 37ºC. Plates were incubated with 25 μL of serum samples diluted in PBS with 2.5% BSA for 1 h at 37ºC, and then washed 3 times with PBS-T, followed by incubation at 37°C for 1 h with 25 μL of the secondary anti-dog IgG conjugated to horseradish peroxidase (1:2,500 in 2.5% PBS-BSA). Plates were washed 3 times with the wash solution, followed by the addition of 50 μL/well of the substrate solution (0.1 M anhydrous citric acid, 0.2 M sodium phosphate, 0.05% o-phenylenediamine dihydrochloride (OPD, P1526-100G, Sigma-Aldrich) and 0.1% H₂O₂, and plates were incubated protected from light for 10 min with the developer solution. Finally, the reaction was stopped by adding 25 μL/well of H_2_SO_4_.

For the iELISA, the adsorption of synthetic peptides on the plate (polystyrene Maxisorp – Nunc) was performed with phosphate-buffered saline (PBS) 1x, pH 7.2 at a concentration of 2.0 μg/well for 18 h at 37°C. Excess of blocking solution was removed by three successive washes. The serum samples were diluted 1:50 in incubation buffer (2.5% BSA), and 100 μL of this solution was applied to each well. Incubation was performed for 60 min at 37ºC, and the excess diluted serum was removed by a series of five washes in 1x PBS with 0.05% Tween 20. The conjugate was diluted 1:2000 and 100 μL of this solution was added to each well. After a new incubation for 60 min at 37ºC, the excess conjugate was removed by a new series of five washes. and 50 μL/well of the substrate was added (0.1 M anhydrous citric acid, 0.2 M sodium phosphate, 0.05% OPD – ortho-phenylenediamine (OPD, Sigma Aldrich, USA) and 0.1% H₂O₂). The reaction was performed for 10 min at room temperature, when it was interrupted by the addition of 25 μL of 4 N sulfuric acid (H_2_SO_4_) (Merck, Germany) per well.

For using the multi-epitope protein as antigen, half area ELISA plates (Corning, USA) were sensitized with 100 μL of phosphate-buffered saline (PBS) 1x, pH 7.2 containing 100 ng/well of protein for 18 h at 4ºC. After protein adsorption, plates were washed 3 times with the wash solution (PBST 0.05% Tween 20) and blocked with 100 μL of PBS1x plus 5% bovine serum albumin (BSA) for 1 h at 37ºC. Blocking solution was removed from wells by aspiration and 25 μL of serum samples diluted 1:100 in PBS with 2.5% BSA, were added to wells and incubated for 1 h at 37ºC. Plates were washed three times with PBS with 0.05% Tween 20, and 25 μL of the secondary antibody diluted 1:2000 in 2.5% PBS-BSA were added to each well and incubated at 37°C for 1 h. Plates were again washed 3 times with the washing solution, and 50 μL/well of the substrate was added (0.1 M anhydrous citric acid, 0.2 M sodium phosphate, 0.05% OPD and 0.1% H₂O₂). Plates were protected from light and incubated for 5 min with the developer solution, and then the reaction was interrupted by the addition of 25 μL of 4 N sulfuric acid (H_2_SO_4_).

Absorbance values were measured in ELISA readers at 492 nm.

### Statistical analysis

Analytical sensitivity and specificity were calculated as true positives/ (true positives + false negatives) and true negatives/ (true negatives + false positives), respectively. Analyses of the ROC (Receptor Operating Characteristic) curve, area under the curve (AUC), and confidence intervals were performed using the GraphPad Prism 8.01 (GraphPad Inc, USA), employing the Wilson/Brown method.

## Results

### Reactivity of dog sera with predicted epitopes in cellulose membranes

**Fig 1:**
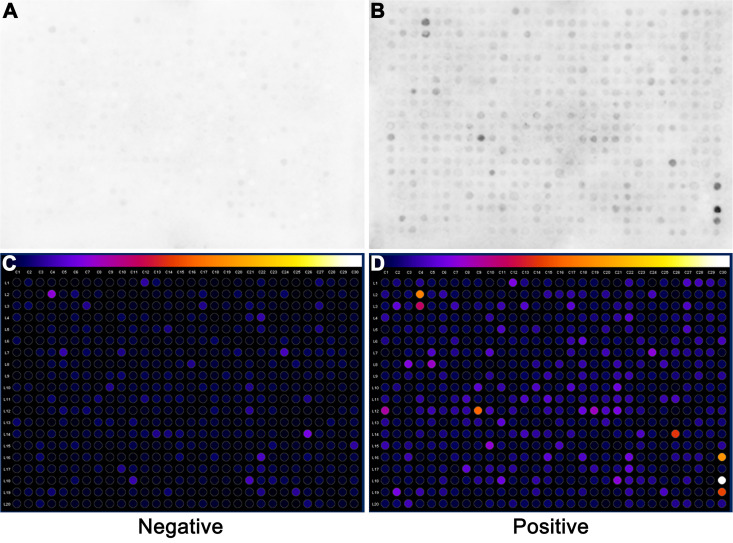
Reactivity of canine sera against membranes containing 542 spotted peptides corresponding to predicted *Brucella* spp. epitopes. Representative images of membranes containing the 542 predicted epitopes incubated with (A) sera from dogs (n = 10) known to be negative for brucellosis or (B) with a pool of sera from dogs (n = 10) known to be positive for canine brucellosis. (C and D) Densitometric analysis of the immunoblots performed using ImageJ – Protein Array Analyzer (NIH, USA), showing the reactivity of canine sera that are negative (A) or positive (B) for *Brucella canis*.

*In silico* prediction of B cell epitopes resulted in the identification of 542 peptides with potential for use in serologic diagnosis of brucellosis. After the epitope prediction analysis, 370 peptides were found to be conserved across all *Brucella* spp. included in this study, whereas 172 peptides were species-specific. All 542 peptides were synthesized and spotted on membranes for immunobloting. Pools of serum samples from dogs known to be positive (n = 10) or negative (n = 10) for *B. canis* were incubated with cellulose membranes containing the predicted peptides. The membranes that were incubated with sera from dogs considered negative for *B. canis* did not have detectable reactivity (Fig 1A), while some spots of the membranes incubated with sera from dogs naturally infected with *B. canis* exhibited higher signal intensity, indicating interaction of some peptides with sera from infected dogs (Fig 1B). Evaluation of the intensity of reactivity was performed using the “Protein Array Analyzer” program of the ImageJ software (NIH, USA), and representative results are in Fig 1C and 1D, which confirmed that negative sera remained without detectable signal (Fig 1C) and positive sera reacted with some of the predicted epitopes (Fig 1D).

### Selection of immunoreactive peptides by densitometric analysis

**Fig 2:**
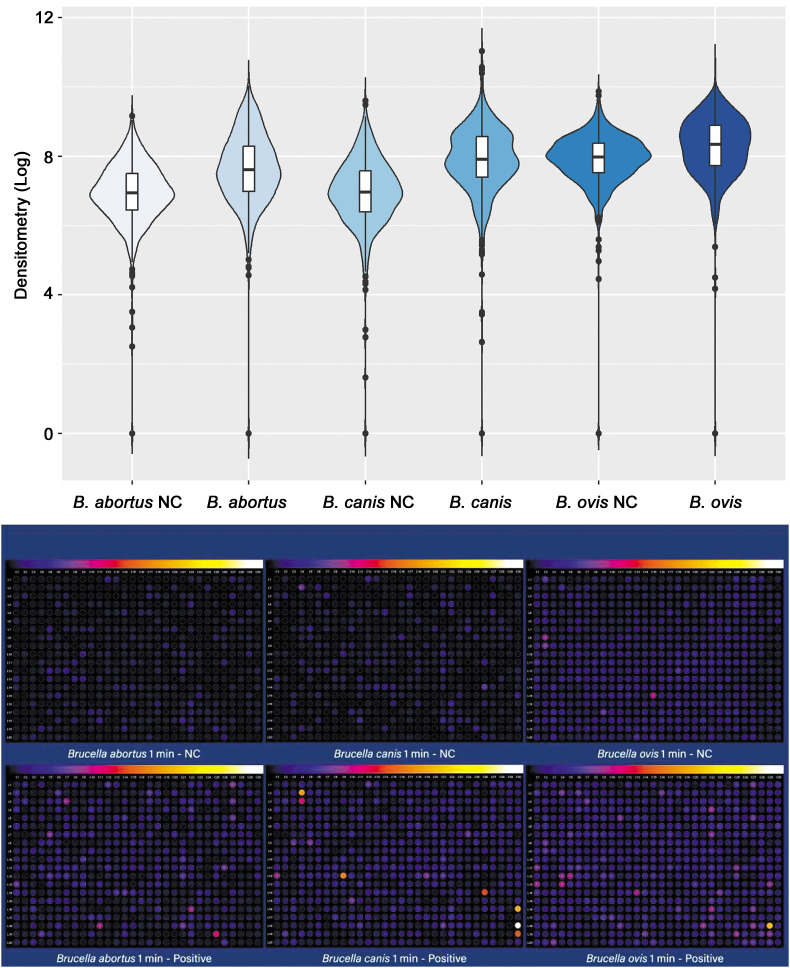
Graphical representation of reactivity of bovine, canine, and ovine serum pools applied on membranes containing predicted *Brucella* spp. B cell epitopes. On the top, graphical representation of the reactivity of pools of bovine, canine, or ovine sera, known to be positive or negative (NC) for *Brucella abortus*, *Brucella canis* and *Brucella ovis*, respectively. On the bottom, representative images of densitometric analysis.

Densitometric analysis was performed for comparing all peptides using pools of seropositive or seronegative samples for *B. canis*, *B. abortus*, and *B. ovis* (Fig 2). From the results obtained in densitometry for *B. canis*, it was possible to identify that some peptides exhibit significantly higher intensity when incubated with positive serum for *B. canis*, while they remain with low intensity in the negative and positive sera of *B. abortus* and *B. ovis* (Fig 2).

### Specific and conserved immunoreactive peptides among *Brucella* species

**Fig 3:**
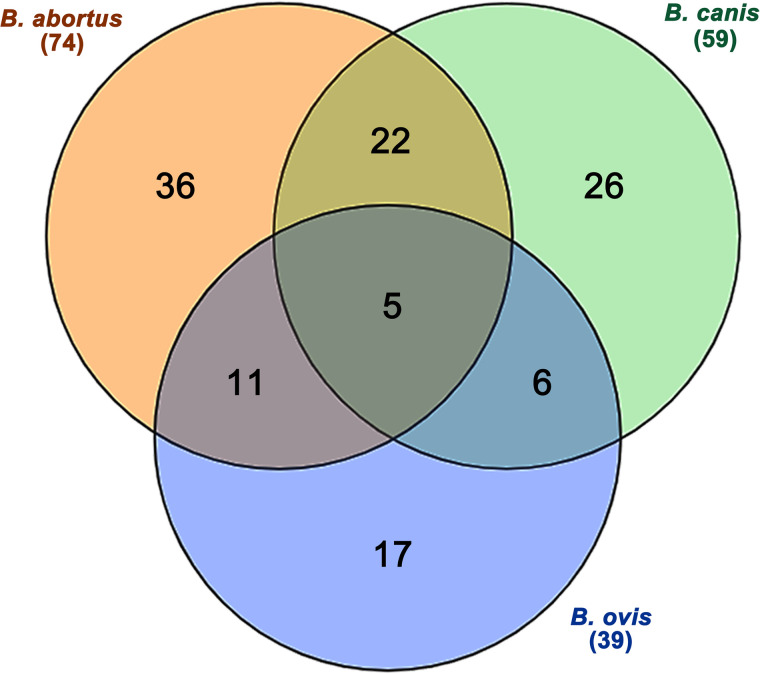
Venn diagram of densitometric analyses of sera reacting with peptides corresponding to B-cell epitopes predicted to be specific to *Brucella abortus*, *Brucella canis*, and *Brucella ovis.* (A) Venn diagram indicating peptides corresponding to selected B-cell epitopes for different *Brucella* spp. after densitometric analysis using ImageJ software of membranes incubated with pool of bovine, canine, and ovine serum samples known to be positive to *B. abortus*, *B. canis*, and *B. ovis*. Cross reactive peptides were identified among *B. abortus*, *B. canis*, and *B. ovis* (n = 5), as well as species-specific peptides for *B. abortus* (n = 36), *B. canis* (n = 26) and *B. ovis* (n = 17). Shared peptides were also observed between *B. abortus* and *B. canis* (n = 22); *B. canis* and *B. ovis* (n = 6); and *B. abortus* and *B. ovis* (n = 11).

The densitometric analysis using the Venn diagram of the peptides allowed the identification of 74 peptides that were predicted to be species-specific for *B. abortus*, 59 for *B. canis*, and 39 for *B. ovis* (Fig 3). Among these, 36 peptides were exclusively reactive to sera from cattle infected with *B. abortus*, 26 were reactive to sera from dogs infected with *B. canis*, and 17 were reactive to sera from sheep infected with *B. ovis*. Furthermore, 5 peptides cross-reacted with all three *Brucella* species, while 22 were cross-reactive between *B. abortus* and *B. canis*, 6 were cross-reactive between *B. canis* and *B. ovis*, and 11 were cross-reactive between *B. abortus* and *B. ovis*. These results confirms a high degree of cross-reacting antigens among the *Brucella* species, but, importantly, this initial screening lead to the identification of 26 peptides that reacted exclusively with sera from dogs naturally infected with *B. canis* (Fig 3) from which the 15 more reactive peptides were selected for synthesis in a soluble form and further analyses.

### Analysis of peptide specificity compared to *Brucella canis* whole antigen extract

**Fig 4:**
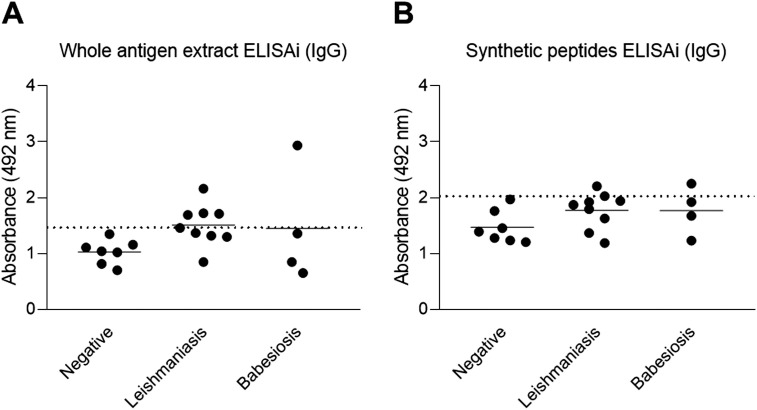
Analytical specificity of iELISA using synthetic peptides for detection of anti-*Brucella canis* IgG. Assessment of the analytical specificity of *B. canis* whole antigen (A) compared to a synthetic peptide-based indirect ELISA (B) for detection of anti-*B. canis* IgG including serum samples from dogs positive for leishmaniasis (n = 9) and babesiosis (n = 4). The dashed line represents the cut-off calculated as the mean of negative controls + 2 x the standard deviation.

After the initial dot blot screening, the most reactive 15 synthetic peptides were synthesized in a soluble form, which were preliminarily evaluated considering their molecular mass and concentrations. Each soluble synthetic peptide was individually evaluated by iELISA using polls of serum samples from *B. canis*-positive or negative dogs. The best 10 synthetic peptides were selected based on their absorbance ratio (positive DO/negative DO) higher than 1.2, and were therefore considered promising for evaluation with individual dog samples (positive, negative and differential) as described in S3 Table. Analytical specificity of selected synthetic peptides was done by comparing the performance of the iELISA with synthetic peptides as antigen against the iELISA using *B. canis* whole antigen extract (crude antigen), applied to serum samples from negative dogs and dogs positive for other diseases including leishmaniasis and babesiosis. Importantly, synthetic peptides had lower cross-reactivity and therefore false-positive results when compared to the crude antigen (Fig 4).

### Synthetic peptides as antigens in a iELISA for detecting anti-*B. canis* IgG and IgM

**Fig 5:**
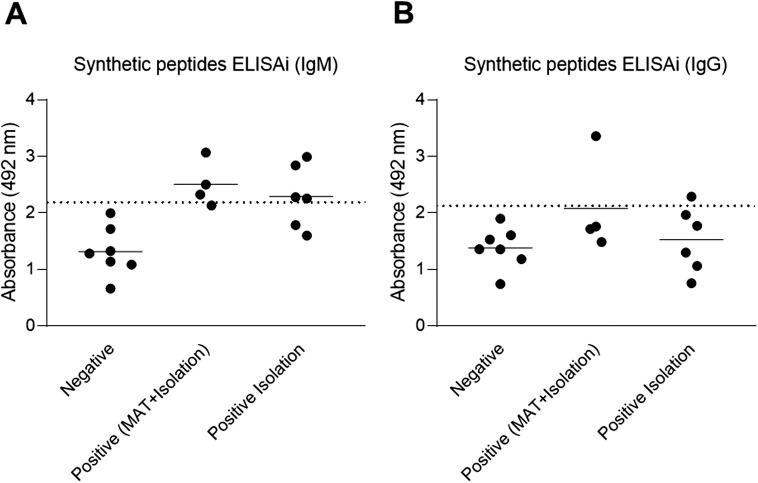
Analytical assessment of iELISA using synthetic peptides for detection of anti-*Brucella canis* IgM and IgG. Detection of anti-*B. canis* IgM (A) and IgG (B) in serum samples from positive (n = 10) and negative (n = 7) dogs using synthetic peptides based on *in silico* prediction of B cell epitopes. Positive samples were obtained from dogs that had bacterial isolation and serologically positive by microagglutination test – MAT (n = 4) or by isolation only (n = 6). The dashed line represents the cut-off calculated as the mean of negative samples + 2 x the standard deviation.

iELISA assays using synthetic peptides as antigens were performed to evaluate their suitability to detect anti-*B. canis* IgM and IgG in positive samples as well as to assess their specificity by evaluating negative control sera. All negative samples remained negative for both IgM and IgG (Fig 5). Assays for detecting IgM reacted in 4 out 5 positive samples (previously characterized by MAT and *B. canis* isolation), while positive samples based only on *B. canis* isolation, resulted in 3 positive results out of 5 serum samples (Fig 5A). IgG detection resulted in identification of 2 out of 5 serum samples from positive dogs (both by MAT and *B. canis* isolation), and all samples positive by *B. canis* isolation only were negative (Fig 5B). Importantly, when results of IgM and IgG are analyzed in combination, analytical specificity and sensitivity were 100% and 80%, respectively, indicating a better analytical performance than the iELISA protocol using the *B. canis* whole extract as antigen. The ROC curve is illustrated in S1 Fig. with an AUC of 0.9 and CI 0.7411 to 1.0 (p = 0.0063).

### Performance of a multi-epitope protein as antigen for serologic diagnosis of canine brucellosis

**Fig 6:**
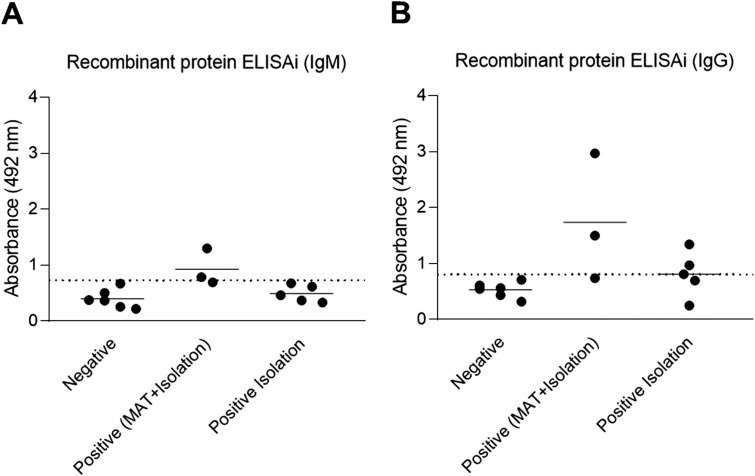
Analytical assessment of iELISA using a multi-epitope protein for detection of anti-*B. canis* IgM and IgG. Detection of anti-*B. canis* IgM (A) and IgG (B) in serum samples from positive (n = 8) and negative (n = 6) dogs using a multi-epitope protein. Positive samples were obtained from dogs that had bacterial isolation and serologically positive by microagglutination test – MAT (n = 3) or by isolation only (n = 5). The dashed line represents the cut-off calculated as the mean of negative samples + 2 x the standard deviation.

A multi-epitope protein containing all 10 peptides (predicted B cell epitopes was expressed in *E. coli* (S2 Fig) and employed as antigen to detect anti-*B. canis* IgM and IgG using positive and negative control samples (Fig 6). However, the number of positive and negative samples available for these analyses decreased slightly since 1 negative and 2 positive samples ran out. Using this antigen to detect IgM all negative samples tested negative, while only 2 out of 8 positive samples tested positive. For IgG detection, all negative samples tested negative, and 5 out of 8 positive samples were correctly identified (Fig 6). These results indicate that the multi-epitope protein had analytical specificity and sensitivity similar to the ELISA performed with synthetic peptides. When results of detection of IgM and IgG are combined, the multi-epitope protein used as antigen yielded analytical specificity and sensitivity of 100% and 75%, respectively. The ROC curve is illustrated in S1 Fig. with an AUC of 0.875 and CI 0.6781 to 1.0 (p = 0.0201).

## Discussion

Serologic diagnosis of canine brucellosis caused by *B. canis* remains a very challenging task due to the limitations of currently available diagnostic tests [3]. In this study a series of synthetic peptides based on *in silico*-predicted *B. canis*-specific B cell epitopes were designed, synthesized, and tested as antigens first trough a immunoblotting screening step followed by their application as antigens in a iELISA protocol. Those same epitopes were inserted into a multi-epitope protein that was expressed in *E. coli* and employed as antigen in an ELISA protocol. These methods resulted in a very high analytical specificity (100%) and good analytical sensitivity (75–80%). This study also clearly indicated that an optimized method for serologic diagnosis of canine brucellosis caused by *B. canis* should be capable of detecting both IgM and IgG. This approach allowed us to prevent cross reactivity with other relevant canine pathogens that may interfere with serologic diagnosis when using *B. ovis* whole extract as antigen. Importantly, currently employed diagnostic methods such as rapid slide agglutination test with 2-mercaptoethanol or AGID have diagnostic sensitivity of 31.76%, and 52.94%, respectively [9], which indicates that in spite of what could be considered limited sensitivity, the methods developed in this study may represent a contribution for improving laboratorial diagnosis of canine brucellosis. Similar results have been obtained by [12], who developed an ELISA assay based on a protein with multiple epitopes that provided high specificity and sensitivity for the diagnosis of human brucellosis caused by *B. melitensis* [12], supporting the concept that *in silico* epitope selection is indeed a powerful tool for developing novel diagnostic methods as demonstrated in this study. Furthermore, [12] applied a similar approach for diagnosing brucellosis in cattle and goats, also demonstrating that the use of proteins constructed from selected peptides increases diagnostic accuracy and reduces cross-reactivity compared to conventional antigens [13]. During the course of this study, a similar *in silico* prediction of epitopes and generation of a multi-epitope antigen was applied to the diagnosis of canine brucellosis, with equally promising results [14].

The genus *Brucella* is highly genetically conserved [19], which may even results in taxonomic confusion [20]. Therefore, identification of suitable species-specific antigens is quite challenging. Yet, in this study we elected to search for species-specific antigens considering the increasing epidemiologic relevant of canine brucellosis due to *B. canis* [2], which is relevant since canine brucellosis due to infections with smooth LPS *Brucella* species have different epidemiological implications [21] and therefore different control measures should be employed. In this study, 26 out of 59 predicted peptides were shown to be indeed *B. canis* species-specific, providing the basis for selection of species-specific antigens potentially useful for differential diagnosis. [22] characterized epitopes of the BP26 protein that specifically reacts with epitopes of a strain of *B. melitensis* [22]. That illustrates how selected epitopes may improve diagnostic application since BP26 is highly conserved among *Brucella* spp. that has been extensively studied as an antigen with potential diagnostic application, with controversial results ranging from a high diagnostic accuracy [23] to poor diagnostic performance [24].

Comparative analysis of synthetic soluble peptides or a multi-epitope protein constructed in this study yielded similar results, indicating that any of these strategies are suitable for generating antigens depending on the availability of scaling for applications in the field. Indeed, previous studies employing multi-epitope proteins resulted in specificity and sensitivity similar to those observed in this study, with high specificity and a suboptimal sensitivity either under laboratory [25] or clinical conditions [26].

Another relevant finding of this study was the superior performance of synthetic peptides compared to crude antigen to prevent cross-reactivity with other canine pathogens, such as *Leishmania* sp. and *Babesia* sp., which are highly prevalent in many parts of the world. Cross-reactivity may lead to false positive results, as observed with the crude *B. canis* extract in this study. The high specificity provided by synthetic peptide antigens or proteins has been previously demonstrated [10]. The methods developed here have potential for diagnostic application in spite of the analytic scope of this study since sensitivity rates demonstrated in this study are higher than those previously reported for currently used tests [9]. In addition, further diagnostic assessment of the methods described in this study may allow excluding the possibility of cross-reactivity with canine pathogens other than *Leishmania* sp. and *Babesia* sp.

This study also demonstrated the need for detection of both IgM and IgG for reaching a higher diagnostic accuracy. Regardless the antigen used (synthetic peptides or multi-epitope protein), there were samples that were negative for detection of IgM and positive for IgG, or vice versa. Therefore, combination of detection of these two immunoglobulin classes increases sensitivity, which is a recommended strategy for the diagnosis of canine brucellosis. Although the profile and kinetics of IgM and IgG production triggered by *Brucella* sp. infection has been quite well studied in the mouse [27], the same does not apply to dogs. Therefore, considering that in clinical settings dealing with natural infections in dogs, it is virtually impossible to precisely establish the time of exposure or infection, and, therefore, attempting to detect both initial and late antibody responses is quite relevant to improve sensitivity.

In conclusion, this study represents a contribution for developing a highly specific test for diagnosis of canine brucellosis caused by *B. canis*. Data on analytical performance of synthetic peptides as well as a multi-epitope protein support further development of protocols based on these antigens, which may contribute to improve diagnostic accuracy of *B. canis* infections in dogs.

## Supporting information

S1 Fig ROC curve analysis of ELISAi results for synthetic peptides and recombinant protein combining IgG and IgM results against B. canis in dogs. (A) ROC curve of iELISA with synthetic peptide for IgM and IgG detection, area under the curve (AUC) = 0.9, 95% confidence interval (0.7411 to 1.000), standard error = 0.08106, p = 0.0063. (B) ROC curve of iELISA with recombinant protein for IgM and IgG detection, AUC = 0.8750, 95% confidence interval (0.6781 to 1.000), standard error = 0.1005, p = 0.0707. Curve generated in GraphPad Prism 8.01 (GraphPad Inc, USA), Wilson/Brown method.
(TIF)

S2 Fig Western blot analysis of the multi-epitope protein. (A) using the anti-His antibody and (B) positive sera pool and anti-dog IgG antibody. The primary antibody anti-His (monoclonal anti-His Tag antibody produced in mouse, GE Healthcare Life Sciences, UK, code 27-4710-01) was diluted 1:1,000 and the secondary anti-Mouse IgG-HRP (Goat Anti-Mouse IgG HRP Conjugate (H + L), Sigma-Aldrich, USA, Cat. No. 71045-M) was diluted 1:3000. Positive sera poll was diluted 1:1000 and secondary anti-IgG canine antibody (Invitrogen, USA, Cat. No. [PA1–29738]) 1:10,000. The multi-epitope protein has an approximate molecular weight of 47 kDa.
(TIF)

S1 Table Genome sequences of *Brucella* spp. employed in this study. (PDF)

S2 Table Optimization of ELISA conditions: antigen concentration, dilution of serum sample, and dilution of the secondary antibody, with the respective signal-to-noise ratio. (PDF)

S3 Table Selected synthetic peptides with the highest absorbance ratio between positive and negative samples (higher than 1.2) as determined by iELISA. These peptides were also incorporated into a multi-epitope protein.
(PDF)
