# Supplementary figures and images for: Development of serologic diagnostic test based on in silico predicted synthetic peptides for Brucella canis in dogs
Source: PLoS One. 2026 Feb 17;21(2):e0342574. doi: 10.1371/journal.pone.0342574 (PMC12912580; doi:10.1371/journal.pone.0342574)

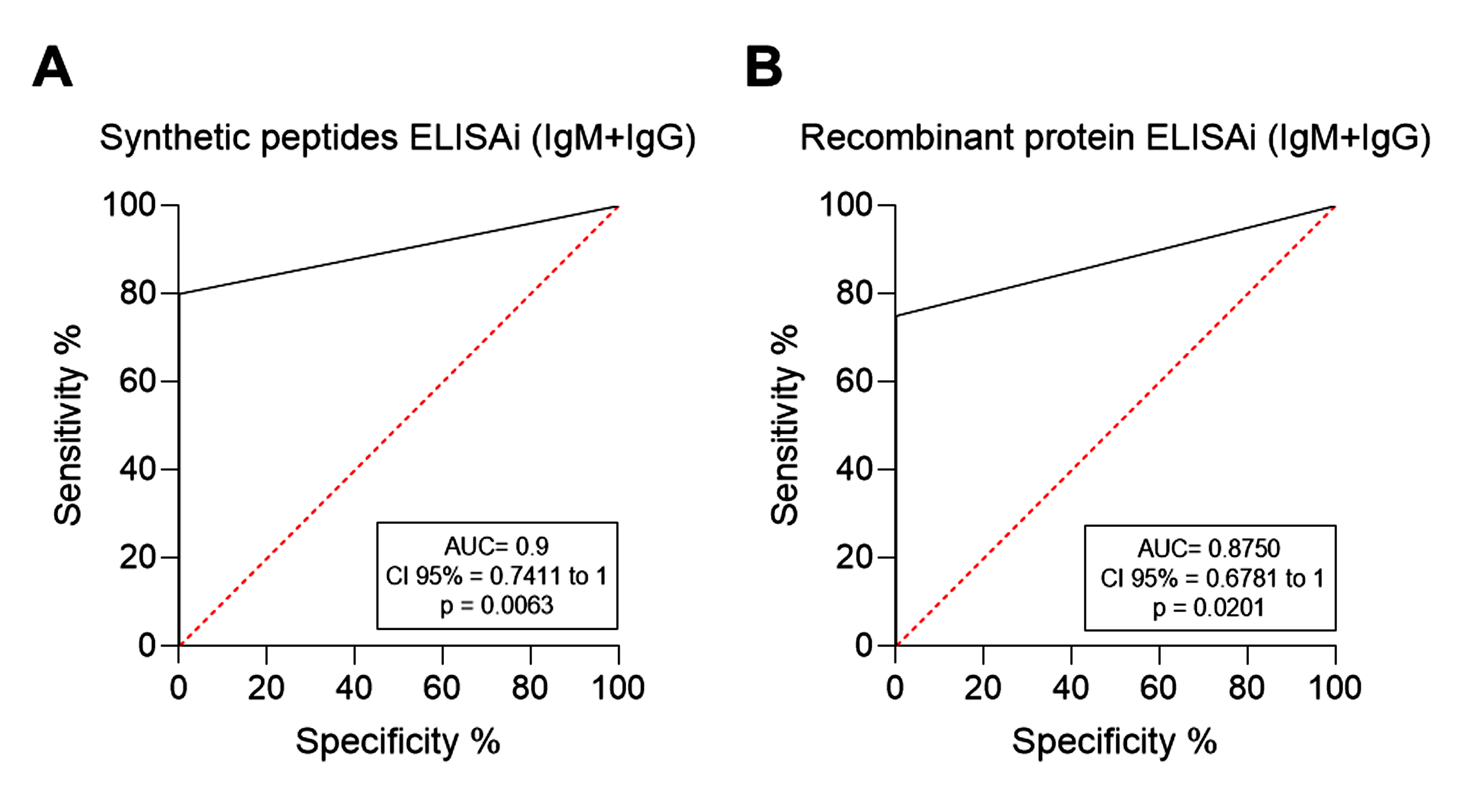

Supplement: S1 Fig — (A) ROC curve of iELISA with synthetic peptide for IgM and IgG detection, area under the curve (AUC) = 0.9, 95% confidence interval (0.7411 to 1.000), standard error = 0.08106, p = 0.0063. (B) ROC curve of iELISA with recombinant protein for IgM and IgG detection, AUC = 0.8750, 95% confidence interval (0.6781 to 1.000), standard error = 0.1005, p = 0.0707. Curve generated in GraphPad Prism 8.01 (GraphPad Inc, USA), Wilson/Brown method. (TIF) [file pone.0342574.s001.tif]

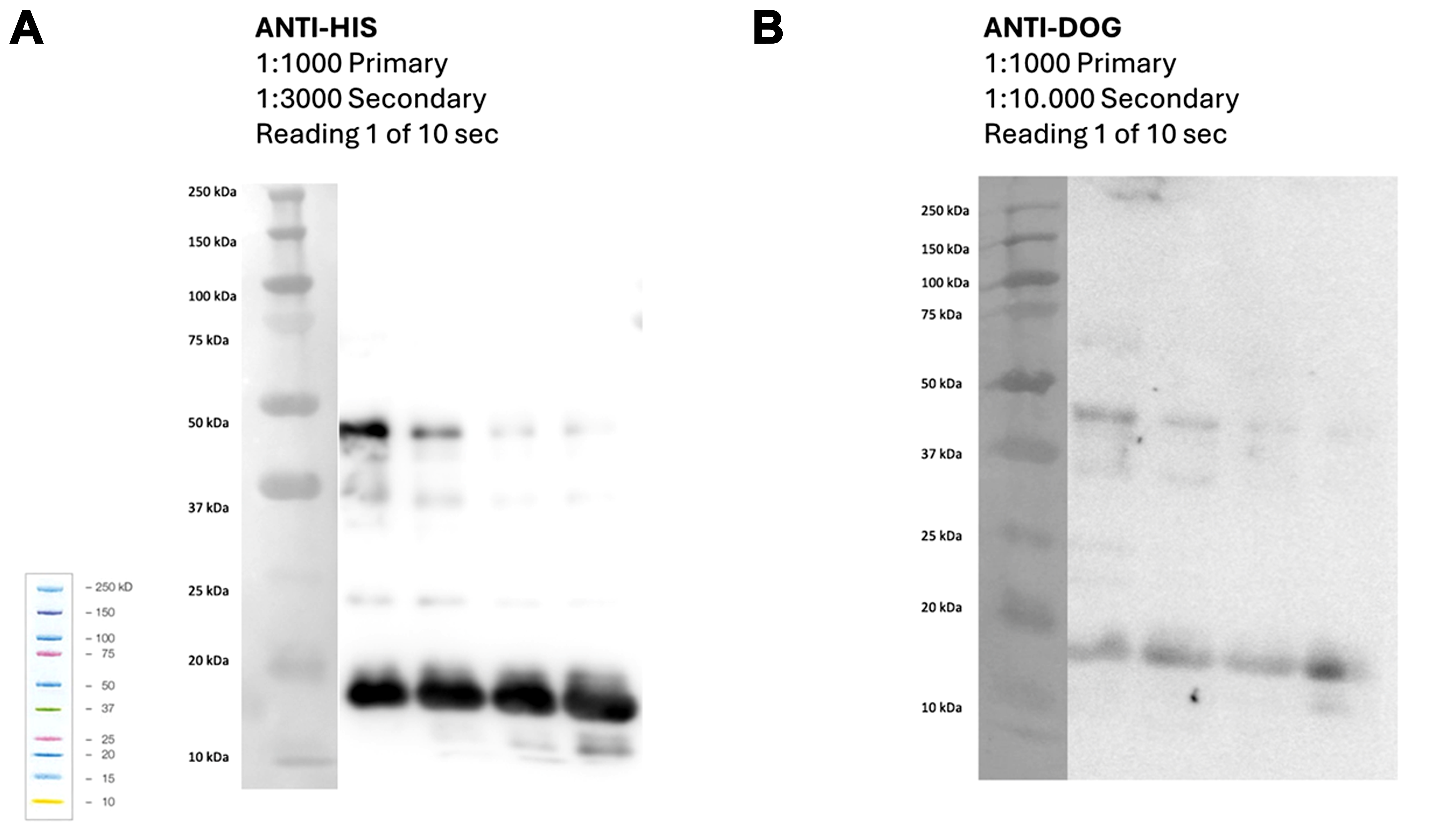

Supplement: S2 Fig — (A) using the anti-His antibody and (B) positive sera pool and anti-dog IgG antibody. The primary antibody anti-His (monoclonal anti-His Tag antibody produced in mouse, GE Healthcare Life Sciences, UK, code 27-4710-01) was diluted 1:1,000 and the secondary anti-Mouse IgG-HRP (Goat Anti-Mouse IgG HRP Conjugate (H + L), Sigma-Aldrich, USA, Cat. No. 71045-M) was diluted 1:3000. Positive sera poll was diluted 1:1000 and secondary anti-IgG canine antibody (Invitrogen, USA, Cat. No. [PA1–29738]) 1:10,000. The multi-epitope protein has an approximate molecular weight of 47 kDa. (TIF) [file pone.0342574.s002.tif]
