# Supplementary material for: Development of serologic diagnostic test based on in silico predicted synthetic peptides for Brucella canis in dogs
Source: PLoS One. 2026 Feb 17;21(2):e0342574. doi: 10.1371/journal.pone.0342574 (PMC12912580; doi:10.1371/journal.pone.0342574)
Supplement: S1 Table — (PDF) [file pone.0342574.s003.pdf]

**S1 Table.** Genome sequences of *Brucella* spp. employed in this study.

| Strain | Predicted proteins | | | Accession codes |
|---|---|---|---|---|
| | Total | Filtered | Ratio (F/T) | |
| *Brucella_abortus*_2308 | 3138 | 2354 | 0.75015 | GCA_000054005.1 |
| *Brucella_suis*_strain_QH05 | 3140 | 2393 | 0.76210 | GCF_002749475.1 |
| *Brucella_canis*_ATCC_23365 | 3145 | 2408 | 0.76565 | GCA_000018525.1 |
| *Brucella_suis*_1330 | 3144 | 2411 | 0.76685 | GCA_000007505.1 |
| *Brucella_melitensis*_bv_1_str_16M | 3090 | 2323 | 0.75177 | GCA_000007125.1 |
| *Brucella_melitensis*_CIT21 | 3125 | 2362 | 0.75584 | GCF_003516045.1 |
| *Brucella_ovis*_ATCC_25840 | 2971 | 2243 | 0.75496 | GCA_000016845.1 |
| *Brucella_abortus*_BJ1 | 3108 | 2338 | 0.75225 | GCF_003675365.1 |
