# Supplementary material for: Development of serologic diagnostic test based on in silico predicted synthetic peptides for Brucella canis in dogs
Source: PLoS One. 2026 Feb 17;21(2):e0342574. doi: 10.1371/journal.pone.0342574 (PMC12912580; doi:10.1371/journal.pone.0342574)
Supplement: S2 Table — (PDF) [file pone.0342574.s004.pdf]

S2 Table. Optimization of ELISA conditions: antigen concentration, dilution of serum sample, and dilution of the secondary antibody, with the respective signal-to-noise ratio.

**Sample**	**Type of Antigen**	**Antigen concentration (µg/well)**	**Serum sample dilution**	**Secondary antibody dilution**	**Mean OD**	**Ratio**
Positive	Peptide	2	1:50	1:2000	1.683	1.546
Negative	Peptide	2	1:50	1:2000	1.088	
Positive	Peptide	2	1:100	1:2000	1.983	1.433
Negative	Peptide	2	1:100	1:2000	1.383	
Positive	Peptide	1	1:50	1:2000	1.191	2.702
Negative	Peptide	1	1:50	1:2000	0.710	
Positive	Peptide	1	1:100	1:2000	1.886	2.655
Negative	Peptide	1	1:100	1:2000	0.710	
Positive	Chimeric protein	2	1:50	1:2000	2.677	1.935
Negative	Chimeric protein	2	1:50	1:2000	1.383	
Positive	Chimeric protein	2	1:100	1:2000	2.606	1,452
Negative	Chimeric protein	2	1:100	1:2000	1,794	
Positive	Chimeric protein	1	1:50	1:2000	2.313	1.973
Negative	Chimeric protein	1	1:50	1:2000	1.172	
Positive	Chimeric protein	1	1:100	1:2000	2.029	2.269
Negative	Chimeric protein	1	1:100	1:2000	0.894	
