# Supplementary material for: Development of serologic diagnostic test based on in silico predicted synthetic peptides for Brucella canis in dogs
Source: PLoS One. 2026 Feb 17;21(2):e0342574. doi: 10.1371/journal.pone.0342574 (PMC12912580; doi:10.1371/journal.pone.0342574)
Supplement: S3 Table — These peptides were also incorporated into a multi-epitope protein. (PDF) [file pone.0342574.s005.pdf]

S3 Table. Selected synthetic peptides with the highest absorbance ratio between positive and negative samples (higher than 1.2) as determined by iELISA. These peptides were also incorporated into a multi-*B. canis* epitope protein.

Peptide	Sequence	Molecular Weight	Ratio
**P1**	NNNNNRKGPNPLSRN	1707,8407	2,231
**P2**	MQAEPEPKQEVKPDP	1721,8188	2,991
**P3**	SDSSSDEGEQGEMDA	1542,5158	2,286
**P4**	GDLNLVNDNPSAVIN	1553,7579	2,549
**P5**	QDNDREEGNDDEAEE	1763,6248	2,272
**P6**	PPGGRRPGSPRRRGE	1630,8780	1,849
**P7**	GRQPREREGRDRRLG	1836,9785	2,229
**P8**	RGNQHRGNQNREEGG	1707,7792	2,974
**P9**	PPRKKTKPSRGAVER	1705,9958	1,958
**P10**	KPVPKPEPKPEEKPT	1699,9403	1,329
